# Alzheimer's disease and its progression reduce pyramidal cell gain and connectivity

**DOI:** 10.1002/alz.70805

**Published:** 2025-10-25

**Authors:** Juliette H. Lanskey, Amirhossein Jafarian, Melek Karadag, Ece Kocagoncu, Rebecca S. Williams, Pranay Yadav, Andrew J. Quinn, Jemma Pitt, Tony Thayanandan, Stephen Lowe, Michael Perkinton, Maarten Timmers, Vanessa Raymont, Krish D. Singh, Mark Woolrich, Anna C. Nobre, Richard N. Henson, James B. Rowe

**Affiliations:** ^1^ MRC Cognition and Brain Sciences Unit University of Cambridge Cambridge UK; ^2^ Department of Clinical Neurosciences and Cambridge University Hospitals NHS Foundation Trust University of Cambridge Cambridge UK; ^3^ Centre for Human Brain Health School of Psychology University of Birmingham Edgbaston Birmingham UK; ^4^ Oxford Centre for Human Brain Activity Wellcome Centre for Integrative Neuroimaging Department of Psychiatry University of Oxford Oxford UK; ^5^ Lilly Research Laboratories Lilly Corporate Center Indianapolis Indiana USA; ^6^ Cambridge Biomedical Campus Neuroscience, BioPharmaceuticals R&D, AstraZeneca Cambridge UK; ^7^ Janssen Pharmaceutica NV, a Johnson & Johnson Company Beerse Belgium; ^8^ Department of Psychiatry University of Oxford Oxford UK; ^9^ School of Psychology Cardiff University Brain Research Imaging Centre, Cardiff University Cardiff UK; ^10^ Department of Psychiatry University of Cambridge Cambridge UK

**Keywords:** canonical microcircuit, connectivity, dementia, dynamic causal modeling, magnetoencephalography, mismatch negativity

## Abstract

**INTRODUCTION:**

Alzheimer's disease (AD) affects neurophysiology by loss of neurons, synapses, and neurotransmitters. A mechanistic understanding of the human disease will facilitate new treatments.

**METHODS:**

Magnetoencephalography was recorded during an auditory mismatch negativity paradigm from healthy adults (*n* = 14) and people with symptomatic AD (*n* = 45, amyloid biomarker positive) at baseline and after 16 months. Fourteen people with AD had repeat magnetoencephalography at 2 weeks to assess test–retest reliability. Dynamic causal models were fitted to the evoked responses and analyzed using parametric empirical Bayes.

**RESULTS:**

Sensor data confirmed that AD and its progression reduce the mismatch negativity amplitude, which had excellent test–retest reliability. Parametric empirical Bayes analyses confirmed that AD progressively reduces extrinsic connectivity between pyramidal cells and superficial pyramidal cell gain modulation.

**DISCUSSION:**

Dynamic causal modeling revealed cellular‐level causes of the neurophysiological deficits observed in AD. This approach may help facilitate experimental medicine studies of candidate treatments.

**Highlights:**

- Magnetoencephalography scanning provides reliable biomarkers that are sensitive to Alzheimer's disease (AD) and its progression, and informative about disease mechanisms underlying cognitive decline.
- In vivo assays of pyramidal cell function during cognitive processes in humans improve our understanding of AD mechanisms.
- The amplitude of the mismatch negativity response is progressively reduced in AD. Reduced pyramidal cell gain and connectivity underlie this neurophysiological deficit. These measures are potential biomarkers for interventional studies.

## 1 BACKGROUND

The development of effective treatments for Alzheimer's disease (AD) remains a major challenge. Such treatments should target disease mechanisms behind the neurophysiological and cognitive consequences of AD.^1^ This requires in vivo assays that quantify the causes of human neurophysiological change.^2^ Generative models of non‐invasive imaging can achieve this with characterization of neural dynamics to the level of cortical layer and cell type from non‐invasive neuroimaging data.^3^

Here, we use generative models with magnetoencephalography (MEG) to test the hypothesis that AD affects pyramidal cell gain and connectivity. We do so in the context of an auditory mismatch negativity paradigm in which neural circuits automatically learn and adapt to changing stimulus frequency. This learning reflects short‐term plasticity and can be achieved by iterative updating of top–down predictions by bottom–up prediction errors.^4^ Prediction errors are weighted by precision, reflecting their reliability.^5^ Disruption to this mechanism may impair signal processing and perception in a noisy environment,^6^ as observed in AD.^6^

The precision of prediction errors is proposed to be encoded by the gain modulation of pyramidal cells in superficial layers of cortex.^5^, ^7^ In rodent models, superficial pyramidal cell gain is modulated by currently licensed cholinergic treatments for AD.^5^ For example, galantamine modulates activity of superficial pyramidal cells, leading to changes in neurophysiological responses to rapidly changing stimuli. Cholinergic agonists and antagonists have opposing dose‐dependent effects on superficial pyramidal cell gain modulation.^7^ The hippocampus, which is involved early in the course of AD, includes cholinergic regulation of superficial pyramidal cells.^8^

Pyramidal cells are directly affected by the molecular hallmarks of AD: extracellular amyloid beta (Aβ) plaques^9^ and intraneuronal tau tangles.^10^ AD pathology correlates with the loss of superficial and deep^11^ and acetylcholinesterase‐rich^12^ pyramidal cells. Tau aggregates develop in pyramidal cells and can spread between pyramidal cells.^10^ Variance in the distribution and burden of tauopathy in AD is closely associated with the clinical phenotype,^13^ cognitive deficit,^14^ and rate of cognitive decline.^15^ The effect of aggregated tau on cognition in part results from synaptic toxicity and dendritic de‐arborization of pyramidal cells.^16^, ^17^

Identification of pyramidal cell function in humans, in vivo, can be achieved by invasive electrocorticography. This is not practical as a basis for research and drug development in the context of AD. An alternative approach is the inversion between functional neuroimaging (e.g., electro/magnetoencephalography [E/MEG]) and biophysical models of the cortex. This approach has been used to study the pathophysiology of non‐AD dementias,^18^, ^19^ and confirmatory studies of drug interventions.^5^, ^18^

Our overarching hypothesis is that the observed physiological effect of AD on the mismatch negativity response is explained by reduced gain modulation of superficial pyramidal cells and reduced effectiveness of extrinsic connectivity between pyramidal cell populations of connected regions. We test two specific predictions: (1) symptomatic AD, in the form of amyloid‐positive mild cognitive impairment and mild AD‐type dementia, is associated with reduced gain modulation of superficial pyramidal cells and reduced extrinsic connectivity between pyramidal cells; (2) the same features of the cortical microcircuit change with disease progression.

## 2 METHODS

We adhered to the Strengthening the Reporting of Observational Studies in Epidemiology (STROBE) guidelines checklist for observational studies.

### 2.1 Participants

Data were drawn from the New Therapeutics in Alzheimer's Disease (NTAD) longitudinal observational study.^20^ The study received a favorable opinion from the East of England Cambridge Central Research Ethics Committee (REC reference 18/EE/0042). Participants gave written informed consent in accordance with the Declaration of Helsinki (1991). The study included control participants with no neurological diagnosis and participants with a clinical diagnosis of AD or mild cognitive impairment (hereafter referred to as people with AD noting that they are both symptomatic and have positive amyloid status) aged between 50 and 85 years. Participants completed (1) amyloid screening through either positron emission tomography (PET) or cerebrospinal fluid (CSF) collection, a positive amyloid status enabled study participation for prospective patient participants; (2) a battery of clinical and neuropsychological assessments; and (3) neuroimaging, comprising magnetic resonance imaging (MRI) and E/MEG protocols. A Mini‐Mental State Examination (MMSE) score > 18 and Clinical Dementia Rating (CDR) of 0.5 or 1 enabled study participation for prospective patient participants and a MMSE score > 24 and CDR of 0 enabled study participation for prospective control participants. People in the control group completed screening and baseline assessments only, while people in the patient group completed longitudinal assessments as follows: clinical and neuropsychological assessments were completed at three time points with 12 to 16 months between sessions; blood collection, E/MEG, and MRI were completed at baseline and again at 12 to 16 months. An additional E/MEG scan was completed 2 weeks after baseline for a subset of people from the patient group.

For the current analysis, we used the MEG, MRI, and demographic baseline data from participants from the Cambridge site. Of the 50 people with AD who completed the baseline MEG scan at the Cambridge site, we excluded two people who did not complete the mismatch negativity task as the earphones did not fit comfortably, two people whose diagnosis was revised during follow‐up, and one due to data recording technical issues at baseline. Of the 15 people who completed the test–retest MEG scans at the Cambridge site, one person was excluded from the analysis because of trigger failures on the retest session. One control participant was amyloid positive and excluded from the current analysis. For the complete list of general exclusion criteria for the NTAD study, please see Table S1 in supporting information. Clinical and cognitive data for participants are given in Table 1.

**RESEARCH IN CONTEXT**

- **Systematic review**: The literature was reviewed using traditional sources (PubMed, meeting abstracts, and presentations) for relevant data relating to pyramidal cells and Alzheimer's disease (AD) in humans, *post mortem* tissue, and animal models. Whereas the effect of AD on pyramidal cell connectivity has been assessed in preclinical models and *post mortem* studies, less was known about its effects on pyramidal cell function in humans, in vivo.
- **Interpretation**: The quantification of pyramidal cell function in people living with AD bridges an important knowledge gap, from bench to bedside. The primary finding of reduced pyramidal cell gain and connectivity is consistent with the preclinical and *post mortem* literature.
- **Future directions**: The article describes a non‐invasive framework for measuring the effects of human disease on different types of brain cell. This can be used to probe human disease mechanisms, and to assess the effect of novel treatments on cellular mechanisms underlying cognitive decline.

**TABLE 1:** Participant demographics and clinical data.

	Controls	A+ AD/MCI baseline	AD/MCI retest	AD/MCI follow‐up	Controls versus AD/MCI (at baseline)
Sex (male:female)	9:5	20:25	6:8	16:16	χ^2^ = 0.98, *p* = 0.32
Handedness (right:left:both)	11:3:0	40:4:1	13:0:1	28:3:1	χ^2^ = 1.86, *p* = 0.40
Baseline age (years)^*^	65.3 (± 7.29)	73.8 (± 7.32)	74.8 (± 7.33)	73.2 (± 7.89)	*t* = –3.80, *p* ≤ 0.001, 95% CI [–13.1, –3.86], BF = 71.67
Baseline education	16.2 (± 3.51)	14.1 (± 3.97)	14.0 (± 4.55)	14.6 (± 4.12)	*t* = 1.88, *p* = 0.07, 95% CI [–0.21, 4.37], BF = 1.02
Baseline CDR^*^	0 (± 0)	0.65 (± 0.23)	0.53 (± 0.23)	0.63 (± 0.23)	χ^2^ = 61, *p* < 0.001
Baseline MMSE^*^	29.4 (± 0.74)	24.8 (± 3.52)	(26.1 (± 3.05)	25.1 (± 3.24)	*t* = 4.75, *p* < 0.001, 95% CI [3.41, 5.66], BF = 1163.50
Baseline ACE‐R^*^	92.4 (± 4.94)	74.2 (± 13.1)	78.5 (± 10.5)	75.7 (± 11.1)	*t* = 5.02, *p* < 0.001, 95% CI [13.4, 22.8], BF = 2722.59
Baseline HIS	0.07 (± 0.27)	0.56 (± 0.8)	0.83 (± 1.03)	0.53 (± 0.82)	χ^2^ = 5.12, *p* = 0.162
Baseline PET (SUVR)^*^	0.98 (± 0)	1.68 (± 0.79)	1.67 (± 0.15)	1.64 (± 0.42)	*t* = –4.18, *p* = 0.04, 95% CI [–0.83, –0.61], BF = 264
Baseline CSF (tau/Aβ1–42)^*^	0.38 (± 0.21)	1.84 (± 0.21)	2.21 (± 0.98)	1.63 (± 0.24)	*t* = –6.33, *p* < 0.001, 95% CI [–2.36, –1.18], BF = 75.8

*Note*: Values are given as mean (standard deviation).

Abbreviations: Aβ, amyloid beta; A+ AD/MCI, people with a diagnosis of amyloid‐positive Alzheimer's disease or mild cognitive impairment; ACE‐R, Addenbrooke's Cognitive Examination revised; BF, Bayes factor; CDR, Clinical Dementia Rating; CI, confidence interval; CSF, cerebrospinal fluid; HIS, Hachinski ischemic score; MMSE, Mini‐Mental State Examination; PET, positron emission tomography; SUVR, standardized uptake value ratio; Yrs, years.

* Denotes significant difference between baseline groups by frequentist and/or Bayesian statistics, as per text.

Sample sizes for patients and controls were justified in Lanskey et al.^20^ Owing to the constraints described above, 45 patients and 14 controls were available for the current analyses. With these samples sizes (and *α* = 0.05), one‐tailed, between‐group *t* tests on behavioral data and evoked response amplitudes can detect effect sizes of > 0.77 with 80% power, while within‐group, one‐tailed *t* tests for the sample of 32 patients with longitudinal data can detect effect sizes of > 0.45 with 80% power.

### 2.2 Task

We used a passive roving mismatch negativity task while the participants watched a muted movie. The auditory mismatch negativity task elicits responses to unexpected “oddball” stimuli followed by rapid plasticity as predictions are updated upon repetition of the new stimulus.^5^ Through non‐magnetic earpieces, the participants hear blocks of short sinusoidal tones binaurally in phase, 60 dB above the average auditory threshold. The tones are presented for 100 ms at 500 ms intervals with frequencies in the range 400 to 800 Hz varying in 50 Hz steps. The frequency of successive tones is the same within blocks but changes between blocks. The number of repeated tones per block varied from 3 to 11. Consequently, the first tone of each block represents a deviant tone, which becomes a new standard tone upon repetition.

### 2.3 Imaging acquisition

MEG data were collected on the ElektaVectorView system and MEGIN Triux Neo scanner. Both scanners were configured with 204 planar gradiometers and 102 magnetometers. The position of five head position indicator coils, standard fiducial points, and > 300 additional head points were recorded using the Polhemus digitization system. Electrocardiogram data were recorded by an electrode on the right clavicle and a second electrode on the left, lower rib. A reference electrode recorded from the left side of the nose and the ground electrode was placed on the left cheek. Electrodes on bilateral canthi and below and above the left eye recorded electro‐oculogram data. T1‐weighted MRI was recorded from each participant with a 3T Siemens PRISMA scanner.

### 2.4 Data analyses

#### 2.4.1 Pre‐processing

MaxFilter version 2.2 software (Elekta Neuromag) was used on the raw data to automatically detect bad channels, perform temporal signal space separation, and correct for head movement. OSL's artefact rejection algorithm (github.com/OHBA‐analysis/osl‐core) was used to remove residual bad channels and trials. Data were down‐sampled to 500 Hz and bandpass filtered between 0.01 and 40 Hz. Independent component analysis was performed using the EEGLAB toolbox^21^ to detect and remove artifactual components that correlated with electro‐oculogram and electrocardiogram timeseries using cardiac spatial and normative blink templates. The residual timeseries were subsequently reconstructed for further preprocessing using SPM12 (r7771). Data were epoched from −100 to 500 ms relative to stimulus onset. Robust averaging was used to average epochs for deviant and repeated trials, with conditions weighted separately. A final low‐pass filter was applied to the data to correct for potential high frequencies introduced during robust averaging.

#### 2.4.2 Sensor space analysis

For the sensor space analysis, planar gradiometers were first combined by calculating their root mean square (RMS) values. We then calculated the mean RMS across planar gradiometers (akin to “global field power”) for each peristimulus time, subject, trial type, and session (baseline, 2 weeks, and annual follow‐up). To estimate the mismatch evoked response, we subtracted the waveform for the deviant tone from that to the first stimulus repetition. To quantify the magnitude of this response, we averaged the mean RMS across our a priori time window of 140 to 160 ms.

The main prediction for the baseline session was that this mismatch response magnitude would be smaller in the patient group than control group, as tested by a one‐tailed, between‐group *t* test. To explore as a function of tone repetition number, we also performed a repeated‐measures analysis of covariance (ANCOVA) with tone repetition number (deviant and repetitions 1:5) as the repeated measure, group (controls vs. patients) for the between‐subject measure, and age as the covariate, correcting for non‐sphericity.

For the longitudinal patient data, the main prediction was that the mismatch response magnitude would be smaller for the annual follow‐up than the baseline session, as assessed with a one‐tailed, dependent‐sample *t* test. To examine the effect of number of tone repetitions, a repeated‐measures analysis of variance (ANOVA) was also performed with tone repetition number and session as repeated measures, correcting for non‐sphericity.

**FIGURE 1:**
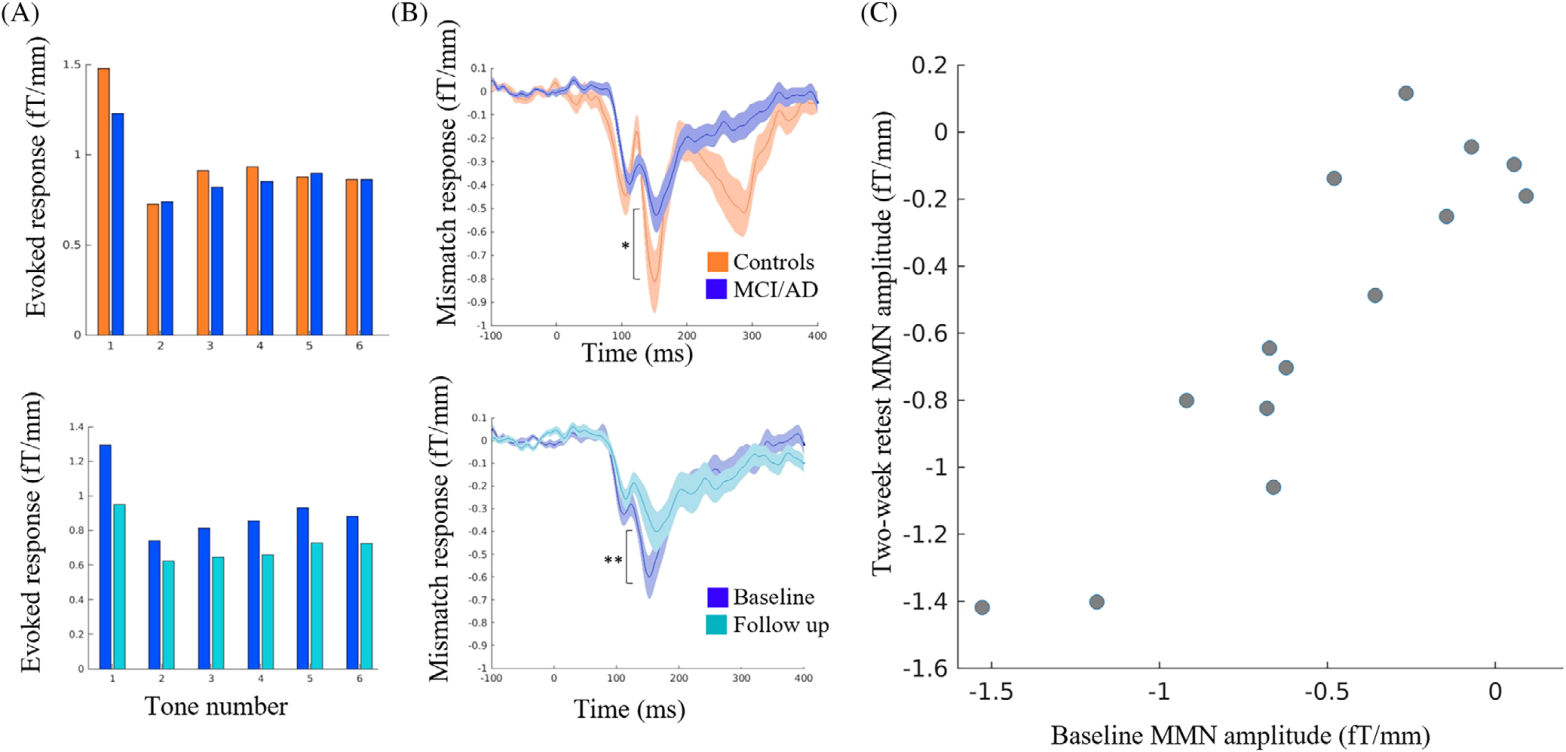
Scalp responses to the mismatch negativity task. A, Amplitude of the evoked scalp responses to tones 1 to 6, averaged across root mean square values of all gradiometers and across samples within the a priori mismatch negativity window from 140 to 160 ms; top panel—for baseline controls in blue and patients in orange; bottom panel—for patients at baseline in dark blue and follow‐up in light blue. B, Average mismatch negativity waveform of repetition 1 minus deviant tones; top panel—for baseline controls in blue and patients in orange; bottom panel—for patients at baseline in dark blue and follow‐up in light blue. C, Absolute intraclass correlation for the mismatch negativity calculated using the test–retest data. MCI/AD, amyloid‐positive mild cognitive impairment or Alzheimer's disease; MMN, mismatch negativity.

For the baseline and 2 week follow‐up data, available in 14 patients, we estimated the test–retest reliability using an absolute, intraclass correlation model (Figure 1).^22^

#### 2.4.3 First level network modeling

**FIGURE 2:**
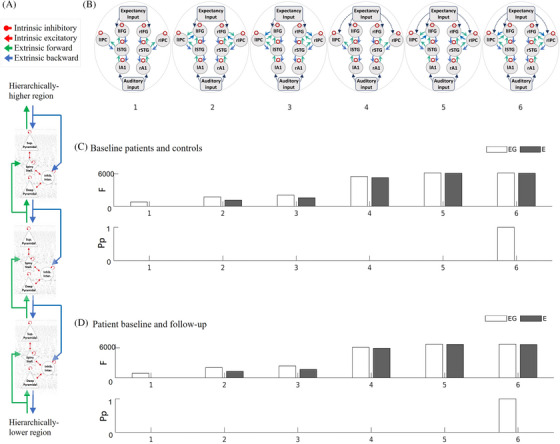
Defining the model structure. A, The canonical microcircuit model for evoked responses. B, Model space for determining the connectivity of the inferior parietal nodes. C, The free energy (top panel) and posterior probability (bottom panel) of models for the patient and control groups; (D) and for the patient baseline and follow‐up groups. A1, primary auditory cortex; E, repetitions varying by exponential decay; EG, repetitions varying by exponential decay combined with phasic change; F, free energy; IFG, inferior frontal gyrus; inhib., inhibitory; inter., interneurons; IPC, inferior parietal cortex; l, left; Pp, posterior probability; r, right; stell., stellate; STG, superior temporal gyrus; Sup, superficial.

We used dynamic causal modeling with laminar‐specific parameters to determine the effect of AD on the neurophysiological generators (Table S2 in supporting information). We used a canonical microcircuit model for evoked responses^23^ comprising four cell populations at each of eight cortical regions (see Figure 2): bilateral primary auditory cortex (left Montreal Neurological Institute [MNI] coordinates: –42, –22, 7; right MNI coordinates: 46, –14, 8), bilateral superior temporal gyrus (left:  –61, –32, +8; right: +59, –25, +8), bilateral inferior frontal gyrus (left: –46, +20, +8; right: +46, +20, +8), and bilateral inferior parietal cortex (left: –58, –27, +30; right: +59, –41, +30). Six of these regions are based on a series of prior studies of mismatch negativity generation with healthy adults and those with non‐AD dementia,^4^, ^18^, ^24^, ^25^ which established a frontotemporal network (primary auditory, superior temporal, and inferior frontal regions) responsible for generating the mismatch negativity response. Other studies have included the parietal cortex in the mismatch negativity network.^26^, ^27^, ^28^ We also extended the frontotemporal network to include the inferior parietal cortex^29^ given the impact of AD on parietal cortex^30^ and its involvement in mismatch negativity generation,^27^, ^29^, ^31^, ^32^, ^33^ with parietal mismatch responses being particularly sensitive to AD.^31^, ^34^ We defined multiple, alternative, symmetric model architectures, each representing plausible alternative hierarchical relationships between regions and their connectivity to include the inferior parietal cortex. Given that higher regions send backward connections to, and receive forward connections from, lower regions in a hierarchical network, we specified forward and backward connections according to the following hierarchy as set out previously:^4^, ^18^, ^24^, ^25^ inferior frontal gyrus > superior temporal gyrus > primary auditory cortex. These connections were unchanging across models. All models included auditory input to primary auditory cortex and expectancy inputs to inferior frontal cortex.^25^ Models 1 and 4 had no connections between frontal and parietal nodes. Models 2 and 5 had the inferior parietal cortex higher than the inferior frontal cortex in the cortical hierarchy. Models 3 and 6 had the inferior frontal cortex higher than parietal cortex in the cortical hierarchy. Models 1 through 3 had no expectancy input to parietal cortex, while models 4 through 6 had expectancy input to parietal cortex (Figure 2B). The laminar asymmetry of forward versus backward connectivity is based on the canonical microcircuit model described by Bastos et al.^23^

We modeled the first six repetitions of tones in the mismatch negativity paradigm to identify the effect of repetition on parameters from the first tone of each block (deviant) to the sixth tone (a new “standard” for each block). In anticipation of short‐term plasticity within the dynamic causal models, we modeled the changes to neural responses over successive tone repetitions as varying by (1) exponential decay or (2) exponential decay combined with phasic change (as used by Garrido et al.^4^). The second set of models with both exponential and phasic change embody the hypothesis of predictive coding for sequential neural responses to stimuli, allowing extrinsic connections to show stimulus‐specific adaptation with exponential decrease with repetition; while intrinsic connections show a phasic response, reducing after the deviant tone and recovering with subsequent tones thereby reflecting the precision of prediction error.^4^, ^24^

The neuronal parameters for each participant's dynamic causal model were inferred from their observed response fields of gradiometers using a lead field informed by subject‐specific T1 magnetic resonance images. Sensor data were modeled from 0 to 300 ms and a Hanning window was applied. This inversion uses the Bayesian variational Laplace method that minimizes the free energy of the model as a cost function to estimate the free energy of the model and posterior estimates of its parameters.^3^ Of the 14 controls and 45 patient participants included at baseline, one control and five patients had an inversion failure due to an integration error during model fitting and were excluded from parametric empirical Bayes (PEB) analyses. For the longitudinal session, three patients failed to invert due to an integration error during model fitting and were excluded from the longitudinal PEB analyses. There was a higher rate of inversion failure for patients (11%) than controls (7%). These algorithm convergence issues may therefore have biased results. While adjusting the priors on parameters or hyperparameters, or adjusting the integration method, may have allowed successful convergence for these subjects, such subject‐specific changes may also have biased results. Moreover, for dynamic causal modeling to be used clinically, we wanted to use its default “out‐of‐the‐box” priors and assumptions. Therefore, excluding data with convergence issues was deemed a safer option.

Bayesian model reduction was applied to each individual's dynamic causal model to identify the most likely explanatory model for group differences (the “winning” model) from the model space. The model space included 12 canonical microcircuit models (Figure 2), with two sets of the six alternative extrinsic connectivity architectures among eight regions; one set describing the effect over sequential tones with an exponential decay and the second set as a combination of exponential and phasic basis functions.^4^ The free energies of reduced models were averaged separately (1) across all participants at baseline and (2) across all baseline and longitudinal data for patient participants. The same model had the highest model evidence for both baseline and longitudinal datasets. This model with the highest model evidence was taken forward to PEB analyses to study group differences.

#### 2.4.4 The effect of AD

Second‐level analysis with PEB of the winning model examined how disease group (healthy control vs. AD) affected parameters modeling the network's response to successive repetitions after a change in tone frequency. We ran two analyses with PEB. For both, the PEB design matrix comprised a unitary regressor (of ones) and a regressor for group membership (zeros for control participants and ones for people with AD). Given a significant difference between groups in age (see Table 1), the baseline PEB analyses were repeated with age as a covariate in the design matrix (see Supplementary Materials in supporting information).

The first PEB analysis tested whether individual differences in the gain of superficial pyramidal cells explains the group difference in the scalp response to the mismatch negativity task. The gain modulation parameter represents a lump sum of features such as potassium conductances and hyperpolarization after an action potential, which act as an auto‐regulation or self‐inhibition of the cell population (rather than being a biological inhibitory connection per se).^5^ A second PEB analysis tested the effect of individual differences in the extrinsic connectivity between pyramidal cells between all regions (both forward and backward connections). For both PEB models, Bayesian model comparison and averaging were performed over a model space of all parameter combinations with hemispheric symmetry and the Bayesian model average results were plotted.

#### 2.4.5 The effect of AD progression

For the analysis of longitudinal change, we performed two second‐level analyses with PEB applied to baseline and annual follow‐up data from patients. Both PEB models included a group mean regressor and a regressor specifying whether the scan was at baseline, given a value of 0, or at the annual follow‐up, given a value of 1. A third regressor specified the time in years between the baseline and follow‐up scans for each patient (0 for baseline scans and with scores from 0.8 to 2.2 for follow‐up scans, mean‐centered and standardized). Two such PEB models were inverted to examine the impact of disease progression on the (1) gain modulation of superficial pyramidal cells and (2) extrinsic connectivity between superficial and deep pyramidal cells. For each PEB model, we performed Bayesian model comparison and averaging over a reduced model space of only those parameters that had differentiated patients versus controls at baseline, and then the Bayesian model average results were plotted.

## 3 RESULTS

Demographic and clinical data are shown in Table 1.

### 3.1 Sensor‐level effects of AD

When averaging over all gradiometers, the amplitude of the mismatch response from 140 to 160 ms was significantly reduced in patients compared to controls at baseline, as predicted (*t* = –1.80, *p* = 0.04, 95% confidence interval [CI] = [–∞, −0.02]). The repeated‐measures ANCOVA on responses to each tone presentation (Figure 1A, upper panel) showed a trend interaction between group and tone presentation number (*F*[5, 280] = 2.48, *p* = 0.08). There was also a significant main effect of presentation number (*F*[5, 280] = 38.0, *p* < 0.001), in which responses decreased dramatically on first repetition (second presentation), and then gradually increased with subsequent presentations, though never to the level of the first (novel) presentation.

For the longitudinal analyses of 32 patients, the mismatch response was further attenuated at annual follow‐up compared to baseline (*t* = –2.72, *p* = 0.005, 95% CI = [–∞, −0.09], Figure 1B lower panel). Consistent with this, the repeated‐measures ANOVA showed a significant interaction between session and tone presentation number (*F*[5, 155] = 16.6, *p* < 0.001). There were also significant main effects of tone repetition (*F*[5, 155] = 20.0, *p* < 0.001) and session (*F*[1, 31] = 75.2, *p* < 0.001).

Finally, analysis of the baseline versus 2 week follow‐up in 13 people with AD showed that the mismatch response had high reliability (intraclass correlation coefficient = 0.95, *p* < 0.001, Figure 1C).

### 3.2 Dynamic causal models

The evoked responses generated by dynamic causal models were accurate, with an average Pearson correlation between generated and observed responses of 0.88 (± 0.17) and 0.84 (± 0.15) for patients at baseline and follow‐up, respectively; and 0.85 (± 0.18) for controls at baseline (see Figures S1–S3 in supporting information).

Model 6 (Figure 2B) had the highest model evidence across both (1) baseline patient and control groups (Figure 2C) and (2) baseline and follow‐up scans for patients (Figure 2D). This model identified the inferior frontal cortex as above the parietal cortex in the network hierarchy. The winning model had the combination of both exponential and phasic basis functions to characterize responses over successive repetitions, in accordance with previous studies,^4^, ^24^ and in line with a predictive coding framework for the mismatch negativity task, rather than stimulus‐specific adaptation only.^4^

### 3.3 Superficial pyramidal cell gain modulation is reduced by AD

**FIGURE 3:**
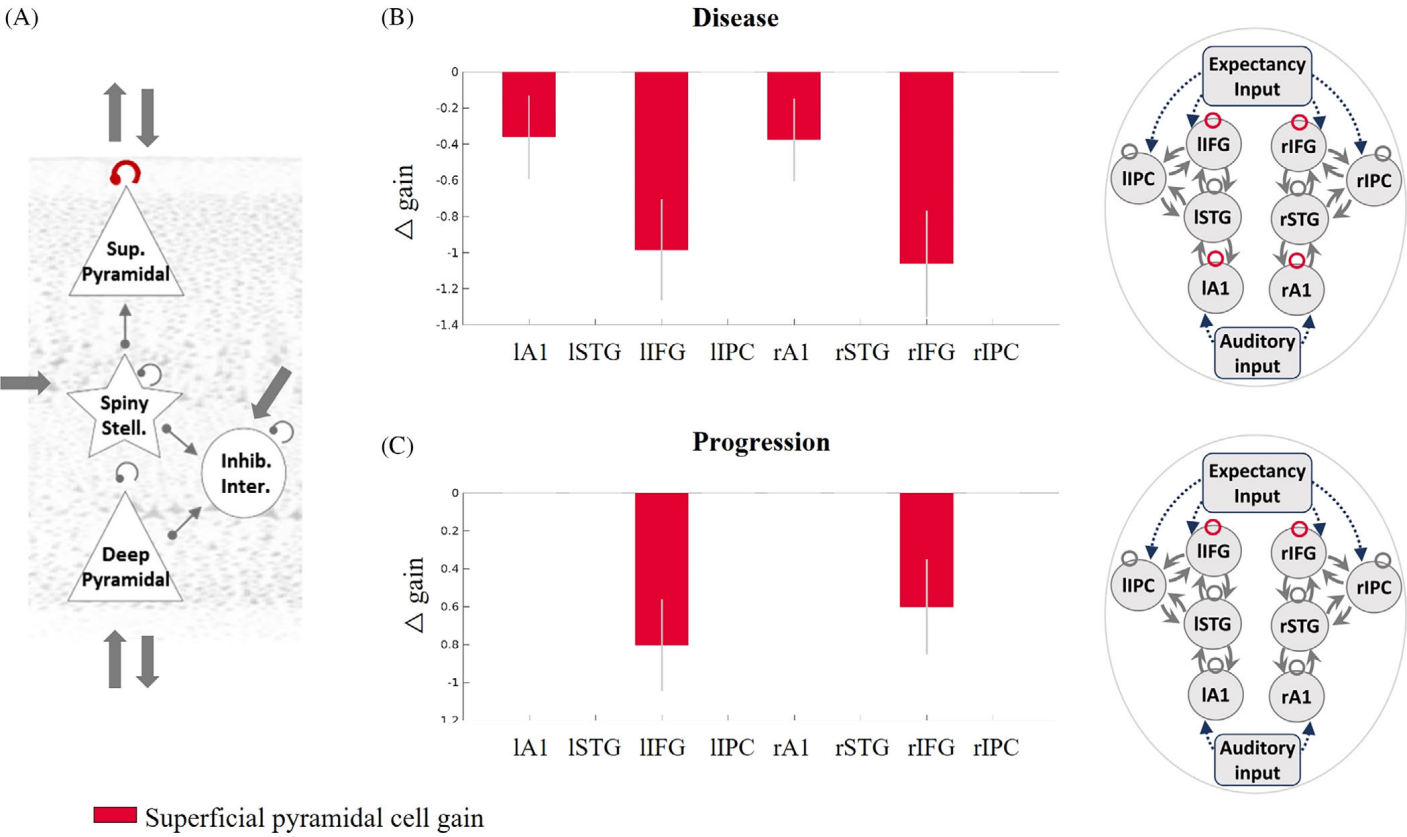
The gain modulation of superficial pyramidal cells and the effect of disease and progression. A, The superficial pyramidal cell gain modulation parameter is shown in red. This parameter was reduced with a posterior probability > 0.95 by (B) disease presence in auditory and inferior frontal regions and by (C) disease progression in inferior frontal regions. The negative direction indicates reduced parameter strength for patients compared to controls. A1, primary auditory cortex; F, free energy; IFG, inferior frontal gyrus; inhib., inhibitory; inter., interneurons; IPC, inferior parietal cortex; l, left; Pp, posterior probability; r, right; stell., stellate; STG, superior temporal gyrus; Sup, superficial.

Analyses confirmed that AD reduced the gain modulation of superficial pyramidal cells in auditory cortices and inferior frontal gyri (Figure 3B). Disease progression also reduced the gain of superficial pyramidal cells in the inferior frontal cortex (Figure 3C).

### 3.4 Extrinsic connectivity: Effect of disease and progression

**FIGURE 4:**
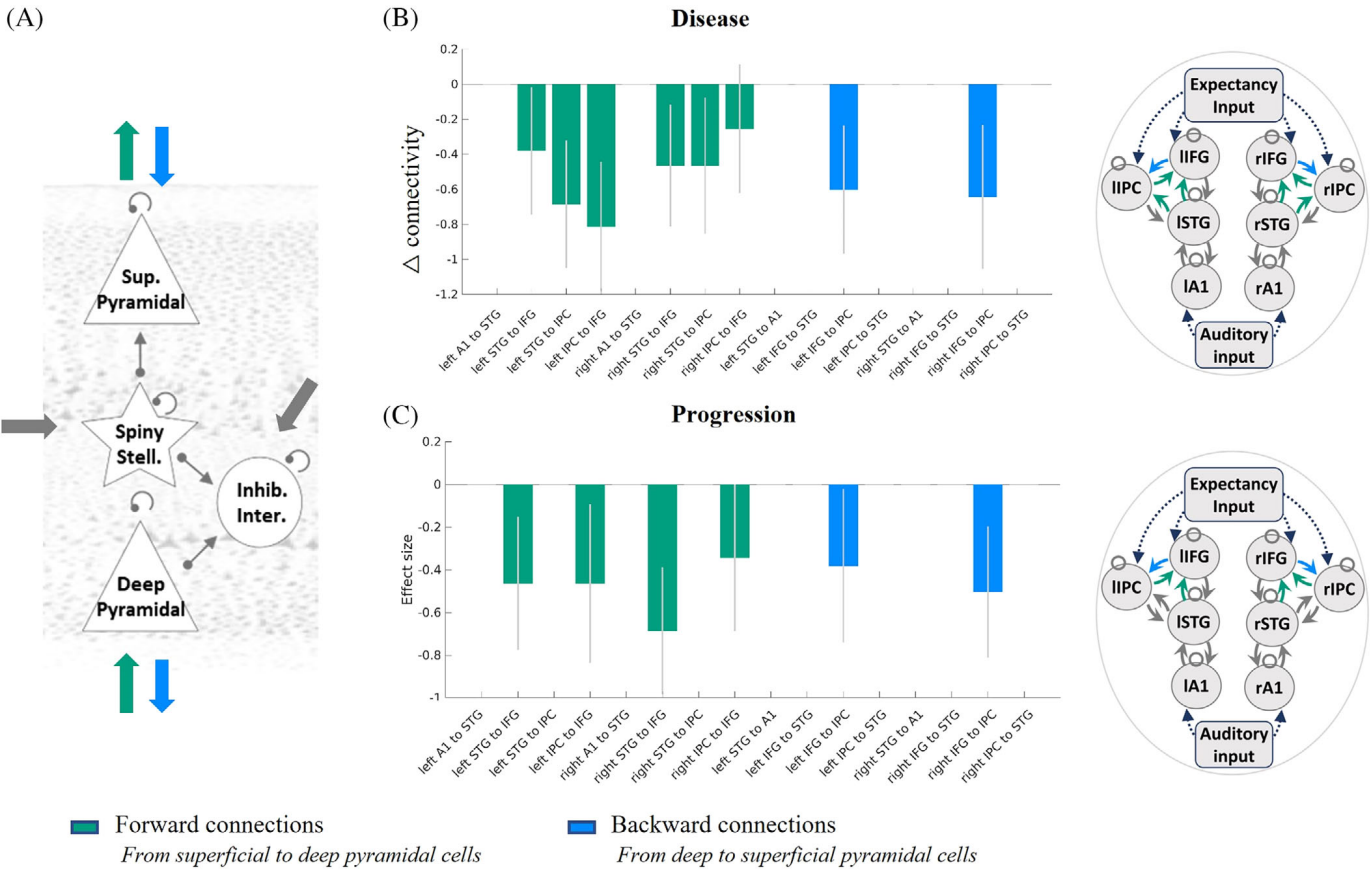
The effect of disease presence and progression on extrinsic superficial and deep pyramidal cell connectivity. A, The extrinsic connections from superficial pyramidal to deep pyramidal cells (green) and deep to superficial pyramidal cells (blue) are shown in the left‐hand diagram. Regions between which these connections were modulated with a posterior probability > 0.95 are shown in green or blue for (B) disease: patient versus controls and (C) progression: baseline versus follow‐up. Alzheimer's disease and its progression reduced extrinsic connectivity between superficial and deep pyramidal cells. A1, primary auditory cortex; F, free energy; IFG, inferior frontal gyrus; inhib., inhibitory; inter., interneurons; IPC, inferior parietal cortex; l, left; Pp, posterior probability; r, right; stell., stellate; STG, superior temporal gyrus; Sup, superficial.

The analyses assessing extrinsic connectivity between superficial and deep pyramidal cells confirmed that the effect of disease was to weaken forward and backward connections. Forward connections were reduced from superior temporal to inferior frontal and inferior parietal cortices, and from inferior parietal to inferior frontal cortices; backward connections were reduced from inferior frontal to inferior parietal cortices (Figure 4B). The effect of disease progression was also to reduce the forward connections from superior temporal to inferior frontal cortices and from inferior parietal to inferior frontal cortices and to reduce backward connections from inferior frontal to inferior parietal cortices (Figure 4C).

## 4 DISCUSSION

This study confirms that magnetoencephalography of a simple auditory paradigm is sensitive to the presence of AD and its progression over 16 months. Furthermore, the mismatch negativity amplitude was highly reliable. Dynamic causal models accurately reproduced scalp responses to the mismatch negativity paradigm. These models revealed the effect of AD on laminar and cellular mechanisms. Critically, we confirmed that the presence and progression of AD reduce pyramidal cell gain and extrinsic connectivity, reducing the mismatch negativity response.

We propose the mismatch negativity response as an early neurocognitive marker of AD. Previous mismatch negativity studies have shown differences between people with AD and controls.^34^, ^35^ Although the mismatch response does not directly probe episodic memory, it is correlated with episodic memory in people at risk of AD^36^, ^37^ and is reduced by fibrillary Aβ in preclinical models.^38^ The mismatch paradigm has important advantages as a potential platform in translational studies and for drug development. For example, it requires minimal training, can be undertaken at any disease stage, and has a direct animal task homologue.

For neurophysiological measures and their biophysical models to be useful in support of experimental medicine studies they must do more than merely differ between people with dementia and healthy adults. They should also be sensitive to disease progression and be interpretable in terms of pathological or pharmacological aspects of disease. In this study we focused on the hypothesized impact of disease on superficial pyramidal cell dynamics and connectivity.

The cholinergic deficit in AD is one mechanism by which cortical generators of the neurophysiological response may differ. For example, mismatch negativity responses are altered by cholinergic antagonists^39^ and by deep brain stimulation of the nucleus basalis of Meynert, a major source of cholinergic innervation of the cortex.^40^ During mismatch negativity paradigms, cholinergic agonists increase^5^, ^7^ and cholinergic antagonists decrease^7^ parameters for the gain modulation of superficial pyramidal cells. We therefore predicted an effect of AD on the gain modulation of superficial pyramidal cells generating mismatch responses. Indeed, AD reduced gain modulation in primary auditory and inferior frontal cortices (Figure 3), where there is a high degree of cholinergic fiber loss.^41^ Superficial pyramidal cell gain modulation is proposed to encode the precision of prediction errors.^5^, ^7^ The hippocampus, affected early in the course of AD, may modulate the precision of prediction errors with cholinergic regulation of regions at the top of the cortical hierarchy (Figure 3 of Barron et al.^8^). Situated at the top of the cortical hierarchy of our model was the inferior frontal cortex, which received expectancy input from external regions, such as the hippocampus, and had reduced gain modulation (Figure 3 and Supplementary Materials Regional Comparisons section).

In the context of the current task, we consider short‐term plasticity to be the basis for updating hierarchical neural beliefs with sensory information. Specifically, the changing magnitude of the mismatch response over successive tone repetitions (Figure 1A) is considered to index the precision (certainty) of prediction error.^4^ This is suggested to encode superficial pyramidal cell gain,^5^, ^7^ which is reduced by AD (Figure 3). As proposed by Garrido et al.,^4^ high precision “surprise” in response to a deviant tone is followed by an initial reduction of precision, which gradually recovers as a new standard tone is learnt. This sequence of events leads to the combined phasic‐plus‐exponential‐decay pattern of the magnitude of responses over successive tones (Figures S4–S6 in supporting information). Changes to short‐term plasticity are an early feature of preclinical models of AD,^42^ occurring prior to cell death^43^ and in proportion to cognitive impairment.^44^ Indeed, we showed reductions in this fast phasic response of intrinsic connections to tone repetition, illustrating an effect of AD on short‐term plasticity (Figure S4).

In addition to the changes within the canonical microcircuit, we illustrate connectivity changes between regions in our large‐scale network of temporal, parietal, and frontal sources. Connectivity changes in large‐scale networks related to AD have been observed with functional MRI and M/EEG.^45^ For example, AD pathology has been associated with connectivity changes to the default‐mode network, frontoparietal network,^46^, ^47^ and networks responding in mismatch paradigms.^48^, ^49^ Pyramidal cells are the principal source of such inter‐regional connectivity. Our analysis suggests that pyramidal cell connectivity is impaired by the presence and progression of AD. Toxic species of aggregated tau affect pyramidal‐cell dendritic complexity^10^, ^16^ even before axonal aggregation.^10^ Tau oligomers and Aβ aggregates are also synaptotoxic.^17^, ^50^ This will reduce sensitivity to incoming connections, reducing effective connectivity.^51^

Another potential contributor to the abnormal mismatch response is synaptic loss and later pyramidal cell death. Transgenic models show disruption of pyramidal cell activity prior to cell death^52^ with significant degeneration of synapses.^53^ Human in vivo studies with synaptic vesicle glycoprotein 2a–binding PET ligands confirm this loss of synaptic density.^54^ From *post mortem* human studies, there is substantial loss of layer 3 (superficial) and layer 5 (deep) pyramidal cells in people with AD compared to controls.^11^ This loss is proportional to the burden of tau neurofibrillary tangles.^11^ This may explain the reduced extrinsic connectivity between superficial and deep pyramidal cells we observed.

For extrinsic connections between superficial and deep pyramidal cells, connections to and from the parietal regions were especially affected (Figure 4B and C). The parietal cortex is commonly implicated in functional and structural imaging of AD.^55^ In contrast, connections to and from primary auditory cortices were not significantly affected (Figure 4 and Figures S7 and S8 in supporting information), in accord with the classical progression of pathology over Braak stages with very late involvement of primary sensory cortices in disease staging.^10^

Different biomarkers vary in their role to understand and quantify the effects of AD. Biomarkers are not necessarily interchangeable, but provide complementary information on diagnostics, mechanisms, progression, severity, or the effects of interventions. They also differ in cost, accessibility, scalability, and vulnerability to known and unknown confounds. For example, effect sizes of group comparisons (people with AD vs. controls) were larger for PET, CSF, and plasma than MEG mismatch negativity biomarkers.^56^ However, PET is much more expensive than MEG while lumbar puncture is more intrusive. Blood biomarkers are more accessible than MEG^57^ but blood and CSF biomarkers provide no information on the localization of pathology or direct information on neurophysiological mechanisms. For localized and mechanistic insights, MEG is exceptional in allowing the application of dynamic models. Furthermore, there were larger effect sizes for longitudinal effects of AD on the MEG mismatch response than for total brain volume or cognitive tests.^56^

Our study has several important limitations. First, our diagnostic groups were defined by clinical diagnoses, albeit supported by amyloid biomarker status. Further work could examine preclinical stages or phenotypic variants. Second, we applied a simplified anatomical model based on earlier studies of the mismatch negativity response augmented by the parietal cortex given its involvement in AD. The model space was defined to suit our hypotheses; however, other models could exist which explain the data. We did not, for example, include the hippocampus and medial temporal cortex, which are affected early in the progression of AD. This is because magnetoencephalographic signals from the hippocampus present methodological and signal‐to‐nose challenges. Specialist techniques, such as rigid head molds or long recording sessions, are not well suited to the patient population. Nonetheless, indirect hippocampal signals may be represented in the form of expectancy inputs to upper layers of the current model. A third consideration is the use of concomitant medication in the patient group (including donepezil, *n* = 17; galantamine, *n* = 1; rivastigmine, *n* = 1; and memantine, *n* = 3). The impact of treatments, such as cholinesterase inhibitors, could therefore have moderated the results; however, participants were required to be on a stable dose for at least 30 days prior to study participation and we would expect cholinesterase inhibitors to reduce (rather than exacerbate) group differences. Fourth, there was a significant difference in age between patient and control groups. However, supplementary PEB analyses which included age as a regressor of non‐interest showed similar results (see Figure S9 in supporting information, where group affected the same parameters in the same direction with age as a covariate). Fifth, attrition at follow‐up could affect results. However, the attrition rate of 29% over an average 16 month interval is in line with previous longitudinal studies. The original study protocol planned for an interval of 12 months, in accord with many early phase clinical trials of disease‐modifying agents. However, the COVID‐19 pandemic lockdowns extended the average interval. Future in vivo human studies could determine whether disease‐modifying treatments targeting the cholinergic deficit or tau depositions in pyramidal cells alleviate the reported findings. Future research can incorporate information about the location of synaptic loss into the model.^19^, ^58^

In conclusion, we have shown how dynamic causal modeling of human neurophysiological non‐invasive recordings is reliable and sensitive to the effects of AD and progression of AD. Specifically, we confirmed changes in pyramidal cell gain and connectivity as predicted by preclinical and *post mortem* studies. This methodology helps to bridge the gap between preclinical and clinical studies, identifying mechanistically informative markers of disease that are sensitive to disease presence and progression.

## CONSENT STATEMENT

All participants provided informed consent in accordance with the Declaration of Helsinki (1991).

## CONFLICT OF INTEREST STATEMENT

M.T. is an employee from Janssen Research & Development, a Division of Janssen Pharmaceutica NV., Beerse, Belgium, and owns stock or stock options in the company. M.P. is employed by AstraZeneca and may currently hold AstraZeneca stocks or stock options. S.L. is employed by Eli Lilly and may currently hold Eli Lilly stock. E.K. is employed by Merck & Co and owns Merck & Co stock options. K.D.S. receives consultancy fees from Draig Therapeutics. M.W. receives FSL royalties and consultancy fees from Wellcome Trust. J.B.R. receives consultancy fees from Astex, Asceneuron, Alector, AstronauTx, Booster Therapeutics, Ferrer, Eisai, ClinicalInk, Prevail, SV Health, Curasen, CumulusNeuro, VesperBio. All other authors have no conflicts of interest. Any author disclosures are available in the Supporting Information.

## Supporting information



Supporting Information

Supporting Information

## Data Availability

The code used is available at https://github.com/jlansk/dcm_cmc_ntad. Anonymized (unlinked) raw data will be made available via Dementias Platform UK, subject to managed access conditions that protect participant confidentiality and conditions of consent.
