# Supplementary material for: Alzheimer's disease and its progression reduce pyramidal cell gain and connectivity
Source: Alzheimers Dement. 2025 Oct 25;21(10):e70805. doi: 10.1002/alz.70805 (PMC12553034; doi:10.1002/alz.70805)
Supplement: Supplementary file 1 — Supporting Information [file ALZ-21-e70805-s001.docx]

# Supplementary materials

| **Group inclusion criteria** | |
| --- | --- |
| **Patients** | **Controls** |
| Diagnosis of MCI or AD | No neurological diagnosis |
| CDR = 0.5 – 1 | CDR = 0 |
| Positive AD biomarker status (CSF or PET) | Known AD biomarker status (CSF or PET) |
| MMSE > 18 | MMSE > 24 |
| 50-85 years | 50-85 years |
| **General exclusion criteria (patients and controls)** | |
| Significant neurological disease, other than Alzheimer’s disease, that may affect cognition or ability to complete the study | |
| Presence of any significant psychiatric disorder that could affect participation ^1^ | |
| Any clinically important abnormality that could compromise study participation | |
| A clinically significant illness, medical or surgical procedure, or trauma within 30 days prior to screening or baseline | |
| Known or suspected systemic infection | |
| Medications affecting cognition, unless on a stable dose for >30 days prior to baseline | |
| Rosen Modified Hachinski Ischaemic score ≥ 4 | |
| History of seizure, except febrile seizures or single provoked seizure | |
| Head trauma resulting in protracted loss of consciousness, or serious infectious disease affecting the brain, within five years of screening and baseline  Participation in a clinical trial of an investigational medicinal product | |
| Impairment of vision or hearing that could affect study participation | |
| Formal education ≤ 7 years | |
| Lack of mental capacity or other ability to consent | |
| Inability to read and write fluently in English | |
| Inability to walk 10 metres independently | |
| Contraindications to blood sampling | |
| Contraindications to lumbar puncture (e.g. spinal deformations) and amyloid PET scan | |
| Contraindication to MRI (including, but not limited to, claustrophobia; pregnancy; MR-incompatible pacemakers and other MR-incompatible, implanted medical devices) | |
| Metallic implants in the body that affect MEG recordings, as judged by the Investigator | |

***Supplementary Table 1 General inclusion and exclusion criteria for participation in the New Therapeutics in Alzheimer’s disease study.*** *MCI=Mild Cognitive Impairment,*

*AD=Alzheimer’s Disease, MMSE=Mini Mental State Examination, CDR=Clinical Dementia Rating. Adapted from Lanskey et al.*^2^ *CC BY 4.0 (*[*https://creativecommons.org/licenses/by/4.0/*](https://creativecommons.org/licenses/by/4.0/)*).*


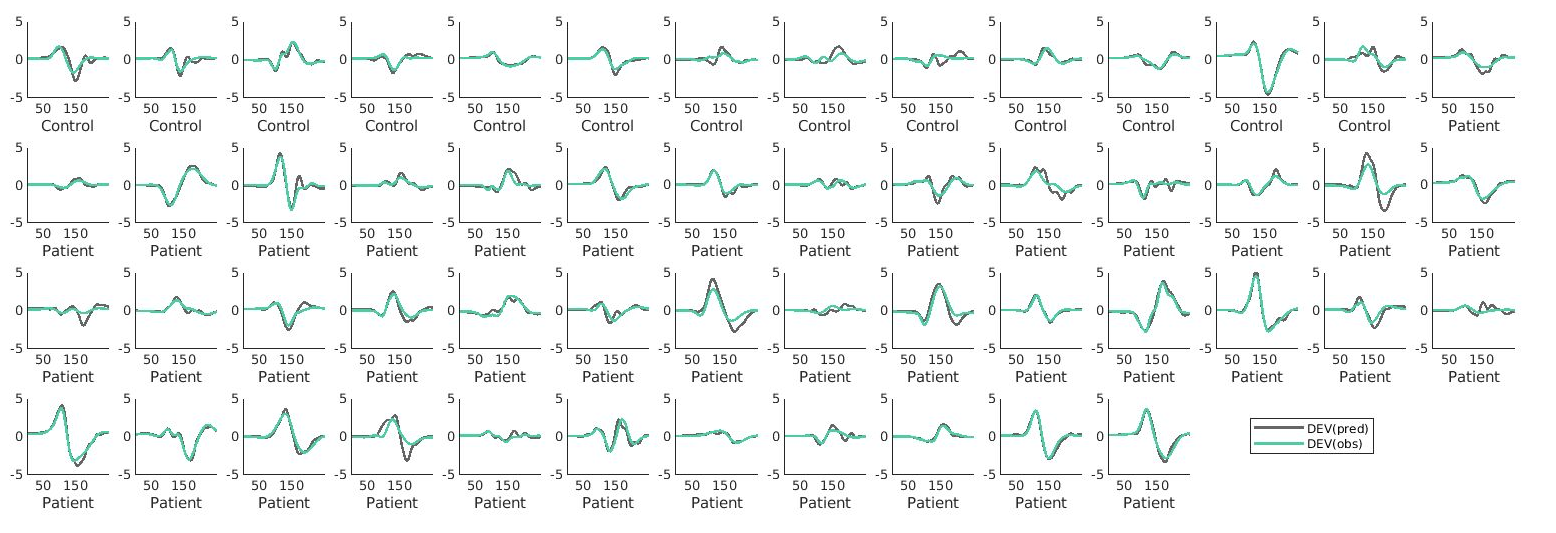


**Supplementary Figure 1 Predicted (grey) and observed (teal) evoked responses to the deviant tone for each subject at baseline**


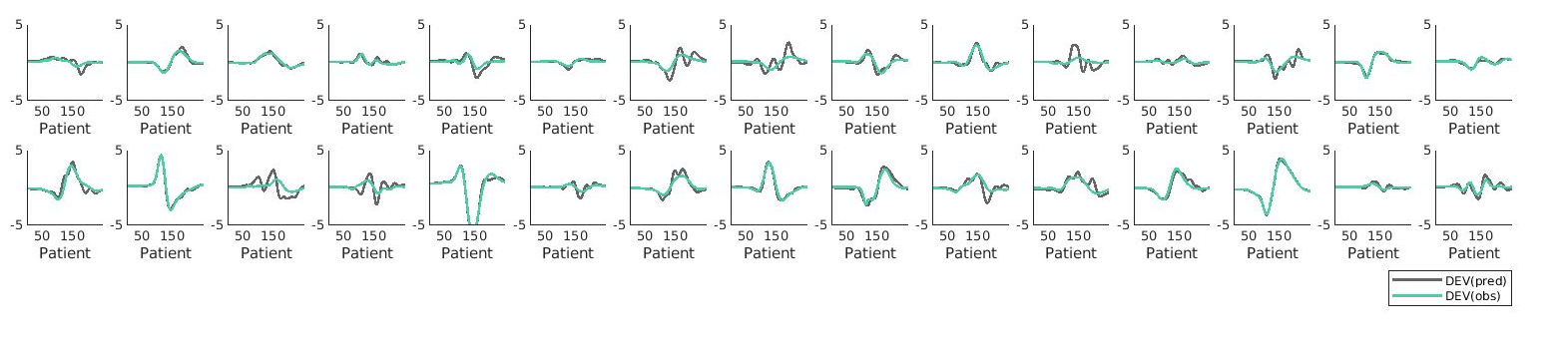


**Supplementary Figure 2 Predicted (grey) and observed (teal) evoked responses to the deviant tone at longitudinal follow up.**


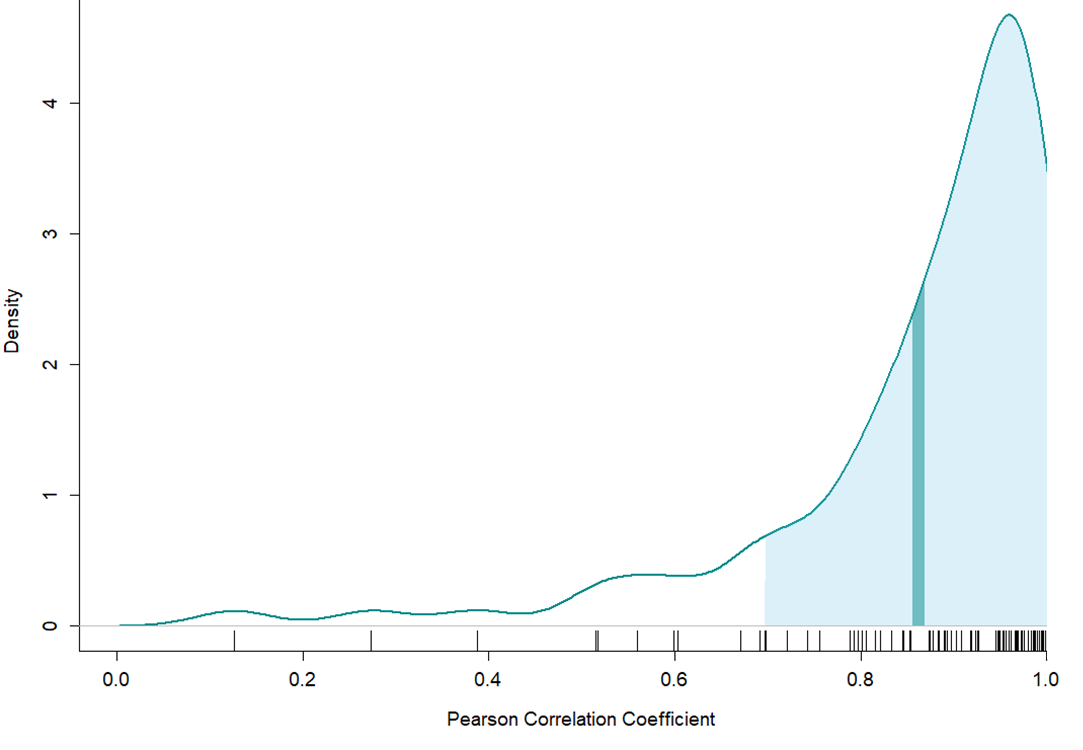


**Supplementary Figure 3 Kernel density distribution of correlation coefficients for observed and predicted evoked responses of all sessions and groups and a rug plot with individual points.**

The Pearson’s correlation coefficient of the predicted and observed evoked responses were calculated for each participant and each session. The kernel density distribution and rug plot of individual correlation coefficients are shown. The observed and model-generated evoked responses were highly correlated (mean Pearson’s correlation = 0.86 ± 0.16).

**Short-term plasticity: effect of disease presence and progression**

To assess the effect of Alzheimer’s disease on short-term plasticity, we inverted two sets of PEB models including parameters for the (i) monotonic (exponential) reduction of forward connections over sequential repetitions; and (ii) phasic repetition effect on intrinsic connections.

To do this, we optimised each set of parameters in PEB models comparing (i) patients and controls, using a design matrix with a first regressor of ones and second regressor of 0 for controls and 1 for patients and (ii) patients at baseline and follow-up, with a first regressor of ones and a second regressor with 0 for baseline DCMs and years-from-baseline for follow-up DCMs. For each PEB model, Bayesian model comparison (function spm_dcm_peb_bmc) was performed over a model space of all parameter combinations with hemispheric symmetry.

We then identified which of the intrinsic parameters (superficial pyramidal cell gain modulation versus intrinsic phasic repetition effect) and extrinsic parameters (connectivity between pyramidal cells versus extrinsic exponential repetition effect) best explained the effect of disease on the neurophysiological responses to the mismatch negativity task (see supplementary figure 8). To do this, we calculated and plotted the difference in free energies for intrinsic (Fig. 3 versus Supplementary Fig. 4) and extrinsic PEB models (Fig. 4 versus Supplementary Fig. 5) of disease presence and progression.

The analyses of sequential stimulus repetition effects reveals how the presence of Alzheimer’s disease changes the phasic effect of stimulus repetition, with a rapid decrease and subsequent increase, in intrinsic connections and slower exponential repetition effect in extrinsic forward connectivity. The phasic response of intrinsic connections was decreased in intrinsic connections of the superior temporal cortices while the slower exponential response was decreased from superior temporal to inferior parietal cortices with an increase from left inferior parietal to inferior frontal cortices (**Supplementary Figure 4**-5).

The PEB models optimising superficial pyramidal cell gain modulation and extrinsic connectivity between pyramidal cells were best at explaining differences between patient and control groups compared to intrinsic and extrinsic repetition effects, respectively (see upper panel of **Supplementary Figure 6**).

There were no significant changes to the effect of repetition with disease progression (patient baseline versus follow up); however, repetition effects on intrinsic and extrinsic connectivity were less important than the effects of superficial pyramidal cell gain modulation and extrinsic connectivity between pyramidal cells for the longitudinal analyses (see lower panel of **Supplementary Figure 6**).


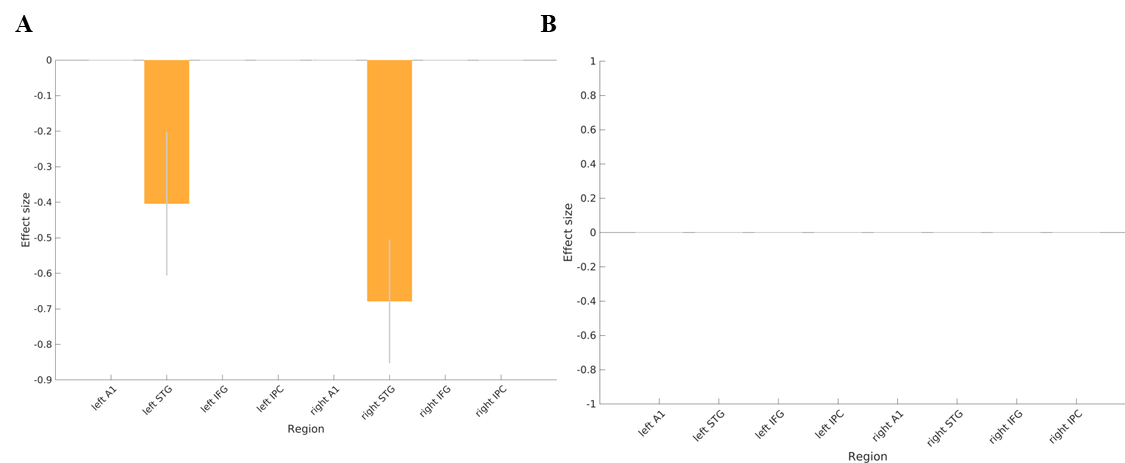


**Supplementary Figure 4** The effect of Alzheimer’s disease and its progression on the intrinsic repetition effect.

The rapid decrease and subsequent increase (phasic) response to tone repetition in intrinsic connections was **(A)** decreased for patients compared to controls; **(B)** had no significant change between baseline and follow up for patients.


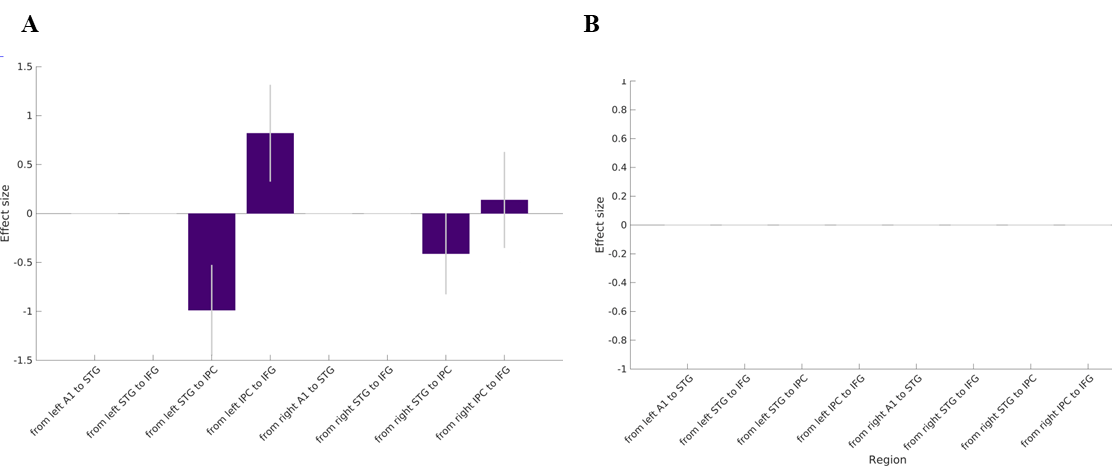


Supplementary Figure 5 The effect of Alzheimer’s disease and its progression on extrinsic repetition effects

**(A)** The slower exponential decreasing response to tone repetition of extrinsic connections was lower in people with Alzheimer’s disease or MCI than controls for the connection from superior temporal to inferior parietal cortices and increased from inferior parietal to frontal cortices. **(B)** There were no significant changes to this response for patients between baseline and follow up.

**
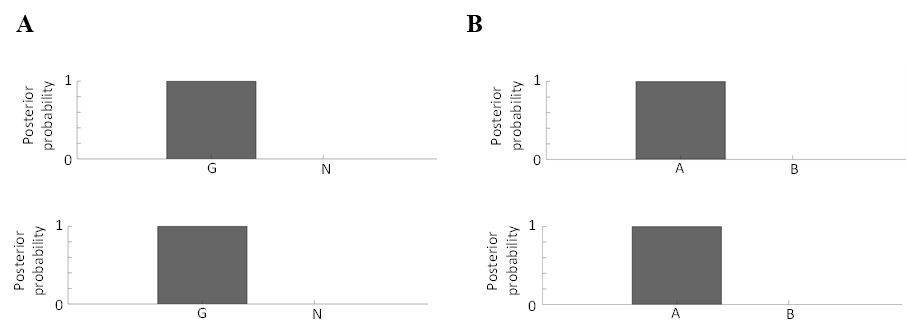
**

**Supplementary Figure 6 Model comparison of PEB models with intrinsic or extrinsic parameters.**

Posterior probability of PEB models for controls versus patients (top) and longitudinally for patients (bottom) with (A) intrinsic parameters (superficial pyramidal cell gain modulation versus phasic repetition effect) and (B) extrinsic parameters (connectivity between pyramidal cells of different regions versus the exponential repetition effect on extrinsic connections).

### Regional comparisons

To identify which regions best explained the observed neurophysiological change with progression of Alzheimer’s disease when considered separately, we compared the free energies of a series of PEB analyses optimising (a) the gain modulation of superficial pyramidal cells and (b) the extrinsic connectivity (between pyramidal cells) of (i) primary auditory (ii) superior temporal (iii) inferior frontal or (iv) inferior parietal nodes.

The gain modulation of superficial pyramidal cells is proposed to index the precision of prediction errors. The hippocampus, affected early in the course of Alzheimer’s disease, is suggested to modulate the precision of prediction errors of regions at the top of the cortical hierarchy (see Fig. 3 of Barron *et al.*^3^). In our model, the inferior frontal region was at the top of the cortical hierarchy and had expectancy inputs, allowing input from external regions such as the hippocampus. For the PEB models optimising the gain modulation of superficial pyramidal cells, the model with inferior frontal regions best described group differences (of the single-region models, see supplementary figure 7)

For the single-region PEB models optimising extrinsic connectivity, the group differences were best explained by the parietal regions (inferior parietal>inferior frontal>superior temporal>primary auditory regions) in accord with the classical progression of pathology over Braak stages.^4^

However, the most likely models incorporated the effects of disease (or follow-up) on all four regions which are therefore reviewed in the main manuscript.


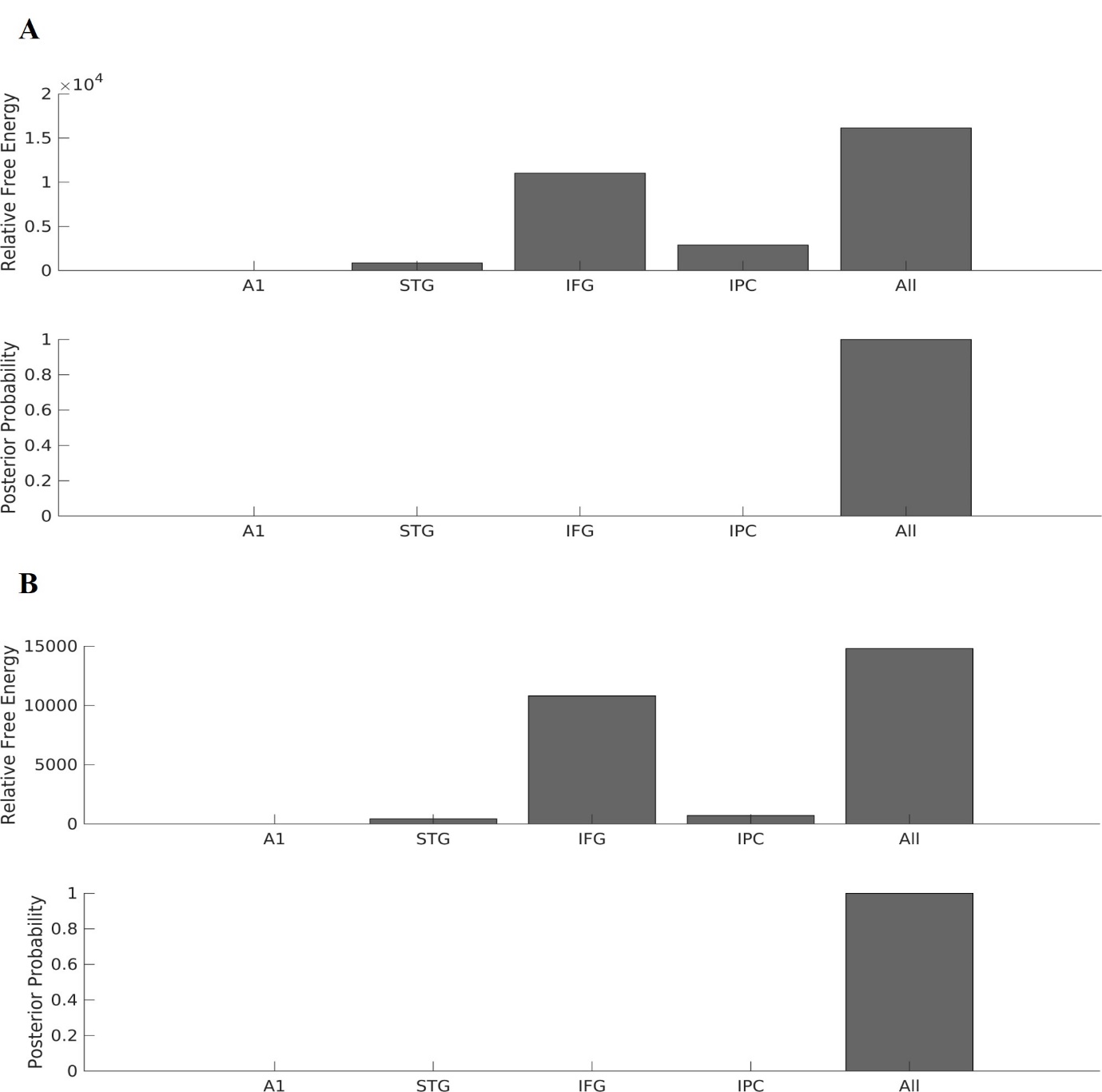


**Supplementary Figure 7 Regional comparisons of PEB models of superficial pyramidal cell gain modulation.**

Regional comparisons of PEB models with (A) controls versus patients and (B) patients at baseline versus follow up are shown. In both instances, for the models including a single region only, the model with frontal regions best explained group differences with a difference in free energy between the frontal and the next highest region, parietal, of 8137 for patient versus control PEB models (A); and 10089 for patient baseline versus follow-up PEB models (B). However, the model with all 4 regions was the winning model, with a difference in free energy between the winning and second-best model of 5121 for baseline patients versus controls (A); and 3998 for patient baseline versus follow up (B). △F, difference in free energy; A1, primary auditory cortices; STG, superior temporal gyri; IFG, inferior frontal gyri; IPC, inferior parietal cortices.


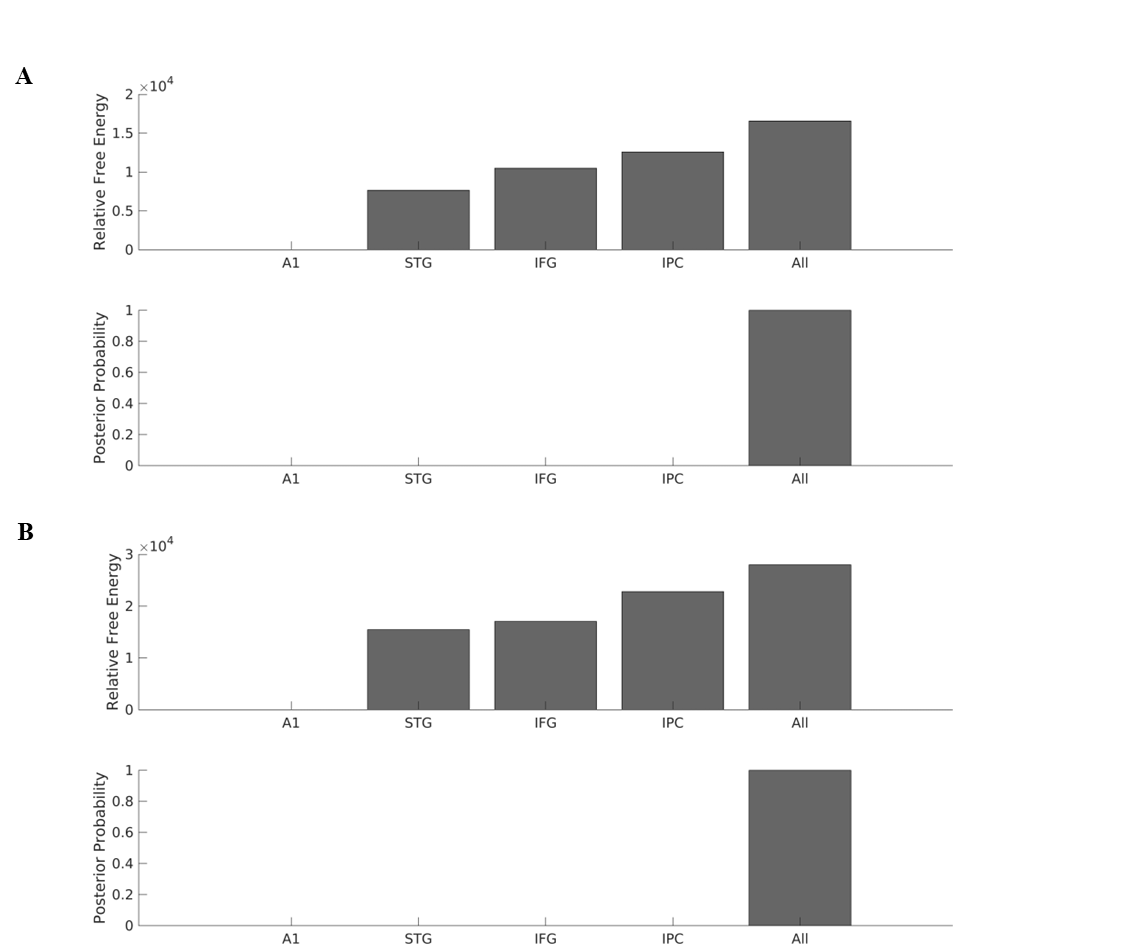


**Supplementary Figure 8 Regional comparisons of PEB models including connections between pyramidal cells of different regions.**

Regional comparisons of PEB models with **(A)** controls versus patients and **(B)** patients at baseline versus follow up. In both instances, for the models including connections originating and terminating from a single region, the parietal model best explained group differences in responses to the mismatch negativity task with a difference in free energy between the parietal and the next highest region, frontal, of 2077 for patient versus control PEBs (A); and of 5770 for patient baseline versus follow-up PEBs (B). However, the model with all 4 regions was the winning model, with a difference in free energy between the winning and second-best model of 3983 for baseline patients versus controls (A); and 5168 for patient baseline versus follow up (B). △F=difference in free energy; A1=primary auditory cortices; STG = superior temporal gyri; IFG=inferior frontal gyri; IPC=inferior parietal cortices.

## Including age as a covariate in the baseline PEB-analyses

Group PEB analyses were inverted with z-scored age as the third regressor in the design matrix. The PEB included all parameters that had differentiated patients from controls at baseline. These analyses were conducted given the significant difference in age between groups. Including age as a covariate had minimal effect on the posterior means and their probabilities of differing between groups (patients versus controls). Age had no significant effect on these connections (see Supplementary Fig. 9).

**Supplementary Figure 9 Group PEB comparisons controlling for age.**

Effects are shown for **controls versus patients (left)** and **age (right)** for (A) superficial pyramidal cell gain and (B) extrinsic connections between pyramidal cells of different regions

# Dynamic causal modelling

In this section, we provide further detail on the biologically-informed generative model of the scalp MEG data used in this paper.

Dynamic casual modelling entails a variational Bayes inversion of biologically-informed models to neuroimaging data under the Laplace assumption (that the priors and posteriors of the unknown parameters have a Gaussian distribution).^5–8^ We use dynamic causal modelling to infer the posterior densities of model parameters and the model’s free energy score from features of the electrophysiological recordings, in this case, the evoked response. The negative free energy of the model provides a lower bound for the log model evidence and is the difference between the predictions of the model’s accuracy and complexity. The objective of variational Laplace is to maximise the free energy score by iteratively updating and adjusting the model parameters given data features and prior information. As the model’s parameters are updated, the accuracy of the predicted model’s response to the observed response is gradually improved, while the inversion scheme penalises model complexity to reduce the risk of overfitting.^9^ The model evidence of different models, with neuronal architectures differing according to the hypothesis being tested, can be compared to elucidate the most likely underlying causes of the observed brain response.

In this study, the neuronal model of each source is the convolution canonical microcircuit model (spm_fx_cmc.m). This model compromises an intrinsic anatomical network in each region, with inhibitory interneurons, superficial and deep pyramidal cells and spiny stellate excitatory populations as shown in Fig. 2A. Specification of the parameters and their priors are set at the default values (spm_cmm_nmda_priors.m). The mean, presynaptic firing rate of each population is scaled by extrinsic connectivity parameters and convolved with a synaptic impulse response function (modelled by single time constant, $T$, with an alpha-shaped kernel, $h\left( t \right)=\frac{t}{T}\exp(-\frac{t}{T})$) to produce the average membrane potential. The mean firing rate (average action potentials) is the sigmoid transformation (denote by $\sigma$) of the membrane potential. Mathematically, the membrane potential in a population $j$ within region $i$, $v_{i}^{\left( j \right)}$, can be calculated by convolving (denoted by$\otimes$) the presynaptic firing rate (the sum of all intrinsic, extrinsic, and experimental firing inputs) with a synaptic kernel as follows:

$v_{i}^{\left( j \right)}=(\underset{\text{intrinsic}}{\underbrace{\sum_{k} \boldsymbol{G}_{ik}^{(j)}\sigma(v_{k}^{(j)})}}+\underset{\text{extrinsic}}{\underbrace{\sum_{kl} \boldsymbol{A}_{jl}^{(ik)}\sigma(v_{k}^{(l)})}}+\underset{\text{experimental}}{\underbrace{\sum_{m} C_{jm}^{(i)}u_{m}}})\otimes h\left( t \right)$ (1)

In equation 1, intrinsic connections $\boldsymbol{G}_{ik}^{(j)}$s (within region $j$, and from population $k$ to $i$) are specified according to laminar-specific features of cortical columns. Each population is subject to self-inhibition, a proxy of self-regulation, to ensure the stability of the model. Extrinsic connectivity, denoted in equation 1 as $\boldsymbol{A}_{jl}^{(ik)}$s (from distal source $k$ to $i$ and from population $l$ to $j$), can be bottom-up (originating from superficial pyramidal cells in a lower level and targeting spiny stellate cells and deep pyramidal cells in a higher level of a cortical hierarchy) and/or top-down (from deep pyramidal cells at high cortical levels to inhibitory interneurons and superficial pyramidal cells in the lower levels).^10,11^ A thalamic drive is denoted by $u_{m}$, which excites spiny stellate cells (in some sources) and models the effect of experimental input (scaled by $C$). The model of an experimental input is a Gaussian function (with latency prior of 70 ± 16 𝑚𝑠).

To generate simulated scalp MEG data, we specified a network of interconnected neuronal sources that are active under the experimental paradigm. The activities of the neuronal sources are approximated as equivalent current dipoles, whose orientation is estimated with symmetry constraints as part of the fitting of the whole model. The models were fitted to sensor data which had been reduced using singular value decomposition to eight principal modes for computational expediency. The generative model is thereby able to predict evoked responses to experimental inputs (in this study, auditory inputs). We fitted this generative model to the scalp MEG data using the variational Laplace scheme in the SPM12 software.

|  | **Description** | **Prior** |
| --- | --- | --- |
| $G_{i}^{(k)}$ | Gain modulation of the *i*-th neuronal population in region *k* | $p(\theta_{G})=N(0,\frac{1}{16})$ |
| $A_{kl}^{(ij)}$ | Extrinsic forward and backward connectivity from population *j* in region *l* to population *i* in region *k* | $p(\theta_{A})=N(0, \frac{1}{8})$ |
| $B_{klm}$ | Change in forward and backward extrinsic connectivity caused by the *m*-th input | $p(\theta_{B})=N(0, \frac{1}{8})$ |
| $N_{jim}$ | Change in intrinsic connectivity caused by the *m*-th input | $p(\theta_{N})=N(0, \frac{1}{8})$ |

**Supplementary Table 2 Parameters of interest from the canonical microcircuit dynamic causal model.**

All parameters used the standard priors and parameterisation (as specified within SPM 12 <https://www.fil.ion.ucl.ac.uk/spm/software/spm12/>). The parameters of interest are shown.

Supplementary material references

1. American Psychiatric Association. *Diagnostic and Statistical Manual of Mental Disorders*. Fifth. American Psychiatric Association; 2013. doi:10.1176/appi.books.9780890425596

2. Lanskey JH, Kocagoncu E, Quinn AJ, et al. New Therapeutics in Alzheimer’s Disease Longitudinal Cohort study (NTAD): study protocol. *BMJ Open*. 2022;12(12):e055135. doi:10.1136/BMJOPEN-2021-055135

3. Barron HC, Auksztulewicz R, Friston K. Prediction and memory: A predictive coding account. *Prog Neurobiol*. 2020;192:101821. doi:10.1016/J.PNEUROBIO.2020.101821

4. Braak H, Tredici K Del. Spreading of tau pathology in sporadic Alzheimer’s disease along cortico-cortical top-down connections. *Cereb Cortex*. 2018;28(9):3372-3384. doi:10.1093/cercor/bhy152

5. Friston KJ, Harrison L, Penny W. Dynamic causal modelling. *Neuroimage*. 2003;19(4):1273-1302. doi:10.1016/S1053-8119(03)00202-7

6. Kiebel SJ, Garrido MI, Friston KJ. Dynamic causal modelling of evoked responses: The role of intrinsic connections. *Neuroimage*. 2007;36(2):332-345. doi:10.1016/j.neuroimage.2007.02.046

7. Friston K, Mattout J, Trujillo-Barreto N, Ashburner J, Penny W. Variational free energy and the Laplace approximation. *Neuroimage*. 2007;34(1):220-234. doi:10.1016/j.neuroimage.2006.08.035

8. Friston KJ, Preller KH, Mathys C, et al. Dynamic causal modelling revisited. *Neuroimage*. 2019;199:730-744. doi:10.1016/J.NEUROIMAGE.2017.02.045

9. Zeidman P, Friston K, Parr T. A primer on Variational Laplace (VL). *Neuroimage*. 2023;279. doi:10.1016/j.neuroimage.2023.120310

10. Felleman DJ, Van Essen DC. Distributed hierarchical processing in the primate cerebral cortex. *Cereb Cortex*. 1991;1(1):1-47. doi:10.1093/cercor/1.1.1

11. Hilgetag CC, Burns GAPC, O’Neill MA, Scannell JW, Young MP. Anatomical connectivity defines the organization of clusters of cortical areas in the macaque monkey and the cat. *Philos Trans R Soc B Biol Sci*. 2000;355(1393):91. doi:10.1098/RSTB.2000.0551
